# Institutional Silence and Informal Coping: A Qualitative Descriptive Study of Nurses’ Experiences of Workplace Violence in Three Hospitals in Riyadh, Saudi Arabia

**DOI:** 10.3390/healthcare14193341

**Published:** 2026-10-07

**Authors:** Abdulaziz M. Alodhailah

**Affiliations:** Department of Medical-Surgical Nursing, College of Nursing, King Saud University, Riyadh 11451, Saudi Arabia; aalodhailah@ksu.edu.sa

**Keywords:** workplace violence, nurses, qualitative research, reflexive thematic analysis, coping, organisational support, post-incident response, Saudi Arabia

## Abstract

Background/Objectives: Saudi evidence on workplace violence (WPV) against nurses is largely survey-based. This study explored how nurses in three Riyadh hospitals experienced and coped with WPV, the institutional factors shaping their responses, and the improvements they proposed. Methods: In this qualitative descriptive study, 17 purposively recruited registered nurses from a tertiary government, a specialised government, and a private hospital took part in face-to-face semi-structured interviews (January–March 2026). Data were analysed using reflexive thematic analysis and reported according to COREQ. Results: The findings describe participants’ experiences and are not prevalence estimates. Four themes were constructed: (1) everyday exposure to violence and its cumulative burden; (2) coping through personal strategies and peer support; (3) institutional silence and gaps in organisational protection; and (4) balancing self-protection and therapeutic care. Participants described relying on personal and peer resources where they perceived institutional protection as insufficient. Their priorities for improvement, reported separately, centred on de-escalation training, simpler reporting, timely post-incident support, and leadership accountability. Conclusions: In participants’ accounts, informal coping absorbed harms that organisational systems did not address. Hospital managers could use these findings to check whether reporting, post-incident follow-up, training, staffing, and security arrangements work in practice. Participants’ proposals require evaluation.

## 1. Introduction

Workplace violence (WPV) against healthcare workers is a global occupational health problem that falls disproportionately on nurses, is widely underreported, and has substantial consequences for those affected [1,2,3]. The International Labour Organization, the International Council of Nurses, the World Health Organization, and Public Services International define WPV as incidents in which staff are abused, threatened, or assaulted in circumstances related to their work, including commuting, involving an explicit or implicit challenge to their safety, well-being, or health [4]. Nurses’ elevated risk has been attributed to sustained patient contact, emotionally charged clinical encounters, and frequent exposure to people in acute distress [5,6].

Reported exposure is high across settings. A review of 136 studies (151,347 nurses) estimated exposure at 36.4% for physical and 66.9% for non-physical violence, with the highest rates of non-physical violence and bullying in studies from the Middle East [7]. More recent meta-analyses report pooled prevalence estimates of 62.3% in African studies [8] and 58% in the South-East Asian and Western Pacific regions [9], and emergency and psychiatric settings are identified as high-risk environments [10]. WPV is associated with burnout, post-traumatic stress symptoms, intention to leave the profession, absenteeism, and reduced quality of care [3,11,12,13].

In the Kingdom of Saudi Arabia, the health sector has undergone rapid reform under Vision 2030 and the National Transformation Program, within a nursing workforce that remains heavily dependent on expatriate staff and faces documented gender-related challenges [14]. This context may introduce distinctive violence dynamics, including language barriers and cultural differences between nurses and patients, hierarchical structures, gender-specific vulnerabilities, and extensive family involvement in patient care [15,16,17]. A recent meta-analysis of 11 Saudi studies (3262 nurses) estimated pooled WPV prevalence at 61.8% (95% CI 45.8–75.6), with verbal abuse most frequently reported [18]. In a national survey of 7398 healthcare workers, 3.9% reported sexual violence at work in the preceding year, and exposure was more common among women, nurses, and night-shift workers [19]; studies in a Saudi university hospital [20] and in primary care centres [21] also report substantial exposure to violence among nurses and other healthcare workers. Most of this evidence, however, derives from cross-sectional surveys using standardised instruments that, by design, cannot capture the meaning nurses attach to violence, the contextual factors shaping their responses, or the practical knowledge they develop through repeated exposure.

This matters for intervention design. Accounts of how nurses make sense of violence, how they experience institutional responses, and what they identify as workable solutions can inform interventions that fit frontline conditions [22,23]. Qualitative research is suited to generating such accounts because it foregrounds participants’ perspectives and retains contextual detail [24,25].

Two theoretical frameworks served as sensitising lenses for the study. Lazarus and Folkman’s transactional model of stress and coping [26] directs attention to how nurses appraise violent encounters and mobilise cognitive and behavioural resources. Hobfoll’s Conservation of Resources (COR) theory [27,28] directs attention to the cumulative loss of psychological, social, and organisational resources under chronic threat, and to the pathway from repeated violence to burnout and attrition. The frameworks informed the scope of the interview guide and were used in the Discussion to interpret the constructed themes; they were not used as a coding frame or to structure the themes (Section 2.7).

Qualitative evidence on nurses’ experiences of WPV has grown internationally, including a systematic review of nurses’ support needs after violent incidents [29], an integrative review of reporting behaviour [30], and primary studies in psychiatric and general hospital settings [31,32]. In Saudi Arabia, qualitative work is limited; it includes a descriptive phenomenological study in a government hospital in the Eastern Region [33] and a study in inpatient mental health facilities [34], and a narrative review concluded that qualitative evidence lags behind survey research [35]. Three gaps follow. First, qualitative accounts drawn from hospitals that differ in ownership and specialisation within the same city appear to be lacking. Second, existing studies concentrate on the forms and consequences of violence and say less about how hospitals respond after incidents and how that response shapes nurses’ coping. Third, little is known about how nurses in this context try to maintain therapeutic care with aggressive patients and families in a workforce where language and cultural differences between nurses and patients are common [15]. Evidence drawn predominantly from other regions may not transfer readily to a health system with different regulatory arrangements, reporting norms, and workforce composition.

Addressing these gaps is necessary for practical as well as scholarly reasons. Because WPV is widely underreported [2], incident records and prevalence surveys show how often violence occurs but not why nurses stay silent, which institutional responses they trust, or where reporting and follow-up break down; hospital managers who rely on such data alone may invest in measures that appear adequate on paper but do not reach the ward. Leaving these questions unanswered has consequences for staff retention and patient care: exposure to violence is associated with burnout, post-traumatic stress symptoms, and turnover intention [3,11,12,13], and in Saudi Arabia these outcomes bear directly on national workforce goals, which depend on retaining experienced expatriate nurses while expanding the Saudi nursing workforce [14]. Prevention and management programmes are also more likely to succeed when they are designed around frontline conditions and nurses’ own judgements of what is feasible [22,23]. The significance of the present study lies in three contributions. First, it provides experience-based evidence from three hospitals that differ in ownership and specialisation within one city, capturing institutional responses across different organisational settings. Second, it traces how post-incident responses shape nurses’ coping, a process that survey data cannot show. Third, it documents nurses’ own priorities for improvement, giving hospital managers and policymakers concrete reference points for reviewing reporting systems, post-incident support, training, and security arrangements.

This study therefore aimed to explore how nurses in three Riyadh hospitals experienced and managed WPV, addressing four objectives: (a) to describe the types and perceived impact of violence encountered; (b) to describe the coping strategies nurses used; (c) to identify institutional and organisational factors that nurses perceived as shaping how violence was managed; and (d) to describe nurses’ own priorities for improvement.

## 2. Materials and Methods

### 2.1. Study Design

A qualitative descriptive design [36] was used, with semi-structured interviews as the data collection method and Braun and Clarke’s reflexive thematic analysis [37,38,39] as the analytic approach. Qualitative description was selected because the objectives called for a contextualised account of nurses’ experiences in terms close to their own, rather than the development of substantive theory (as in grounded theory) or the elucidation of essential structures of lived experience (as in phenomenology) [24,36]. Reflexive thematic analysis is theoretically flexible and can be used within a qualitative descriptive design provided that its theoretical assumptions are made explicit [40]. The study adopted a contextualist orientation: participants’ accounts were treated as meaningful reports of their experiences, while recognising that these accounts are shaped by the social, cultural, and institutional settings in which they occur and by the researcher’s interpretive engagement [37,39]. Consistent with qualitative description, most codes stayed close to participants’ own language (semantic codes), and a smaller number of latent codes captured patterns across accounts (Section 2.7). Interpretations that go beyond participants’ accounts are identified as such in Section 4. The analysis therefore aimed primarily at a descriptive account close to participants’ language, with a limited interpretive layer. Theme names such as “institutional silence” are the author’s summary labels for patterns that participants described in their own words (for example, receiving no follow-up after incidents), whereas concepts such as institutional betrayal and resource loss are theoretical readings applied in the Discussion rather than findings. The study is reported in accordance with the Consolidated Criteria for Reporting Qualitative Research (COREQ) [41]; the completed 32-item checklist is provided as File S1.

### 2.2. Setting

Data were collected at three hospitals in Riyadh, Saudi Arabia, selected purposively to include contrasting organisational contexts within one city. The selection criteria were ownership (government or private), service profile (general or specialised), and patient acuity, because hospitals that differ in these characteristics may also differ in staffing models, patient populations, and the governance and implementation of violence-prevention policy. Riyadh was chosen because it contains government and private, general and specialised hospitals, allowing contrasting sites to be included within one city. The three sites were (i) a large tertiary government hospital serving as a regional referral centre; (ii) a government specialised hospital providing psychiatric, oncological, and rehabilitation services; and (iii) a private multi-specialty hospital. All three hospitals had more than 500 beds (Table 1). Other hospitals in Riyadh share some of these characteristics; the aim was not to represent all hospital types but to include variation in ownership and specialisation. Hospitals are not named, to protect participant confidentiality. For the same reason, participant characteristics are reported by facility category (general or specialised) rather than by site: the tertiary government hospital and the private multi-specialty hospital are both classified as general hospitals, and the government specialised hospital as specialised (Table 2). Sites were not compared analytically.

### 2.3. Participants and Sampling

Seventeen registered nurses (*n* = 17) were recruited through purposive sampling, which enables deliberate selection of information-rich cases [42], supplemented by snowball referral. Inclusion criteria were: (a) current employment as a registered nurse at a participating hospital for at least six months; (b) direct clinical patient-care responsibilities; (c) self-reported experience of, or direct witnessing of, at least one WPV incident; (d) ability to communicate in Arabic or English; and (e) provision of voluntary written informed consent. Nurses in exclusively administrative roles, and those experiencing acute personal crises (identified through initial screening conversations), were excluded.

Recruitment proceeded through three complementary channels: liaison with unit nurse managers, who disseminated study information without coercion; informational posters in staff break rooms; and referral from participants already enrolled. To minimise power differentials, all recruitment materials emphasised voluntariness, and no direct supervisor was present during consent or interview.

A total of 25 eligible nurses were approached across the three sites. Eight declined, citing work-schedule and shift-related constraints, and 17 consented and were interviewed. No participant withdrew after providing consent, and all 17 interviews were completed and included in the analysis.

Sample size was guided by the concept of information power [43]. The final sample of 17 was judged adequate given the focused study aims, the specificity of the sample (nurses who had experienced or witnessed WPV), the depth of the interviews (mean 38 min), and an analytic strategy centred on patterns across accounts rather than on individual cases. The developers of reflexive thematic analysis have argued that data saturation is conceptually incompatible with an approach in which meaning is generated rather than discovered [44]; saturation is therefore not claimed here. It is recorded descriptively that coding of successive transcripts yielded no codes that were not already represented in the coding framework after Interview 15, and that two further interviews (P16, P17) were conducted; this observation informed, but did not determine, the decision to conclude recruitment [45]. The study was not designed to describe each hospital separately or to compare hospitals, and the sample was not sized to achieve adequacy within each site. Recruitment across three contrasting hospitals was intended to broaden the range of experiences represented in a single analysis (Table 1).

### 2.4. Data Collection

Individual, semi-structured, face-to-face interviews were conducted between January and March 2026 by the author, a male nurse researcher who holds a doctoral qualification in nursing and has formal training in qualitative interviewing. The author conducted all interviews alone. He had no pre-existing clinical or supervisory relationship with any participant and was not employed by any of the participating hospitals. At the start of each interview, he introduced himself as a nurse academic at King Saud University and explained that the study was independent of participants’ hospitals and managers (File S2).

An interview guide was developed by the author, informed by the study’s theoretical frameworks, a scoping search of the WPV literature, and consultation with two senior clinical nurse specialists. A short demographic section preceded the interview questions and supplied the participant characteristics reported in Table 2. The guide then comprised 20 open-ended questions across five domains: (i) general violence experiences (e.g., “Can you describe your experience facing workplace violence?”); (ii) violence management strategies (e.g., “What strategies do you use to protect yourself when facing violence?”); (iii) influencing factors (e.g., “How does institutional support affect your sense of safety?”); (iv) interaction with patients and families (e.g., “What challenges do you face when communicating with aggressive patients?”); and (v) development and improvement (e.g., “What recommendations would you make to improve the work environment and reduce violence?”). Probes (e.g., “Can you tell me more about that?”, “How did that make you feel?”) were used flexibly. The guide did not specify who the perpetrator of violence was, so participants could describe violence from patients, relatives, visitors, colleagues, or supervisors. The full guide in Arabic and English is provided as File S2.

The guide was piloted with two nurses who met the eligibility criteria but were not among the 25 nurses approached for the main study, and was refined for clarity and flow; their data are not included in the analysis. Interviews lasted 28 to 52 min (mean 38 min) and were conducted in private rooms within or near the participants’ hospitals, with no one else present. All interviews were audio-recorded with participants’ explicit consent using an encrypted digital recorder. Field notes recording contextual observations, non-verbal cues, and the author’s reflections were completed immediately after each session. No repeat interviews were conducted.

### 2.5. Language and Translation

Of the 17 interviews, 10 were conducted in Arabic and 7 in English. The interview guide was prepared in both Arabic and English. Interviews were transcribed and analysed in the language in which they were conducted, so that meaning and context were preserved in participants’ own words. Quotations reproduced in Section 3 were translated from Arabic into English by the author, who is bilingual in Arabic and English and conducted all interviews, with attention to preserving the original meaning and context of each account. No formal forward and back-translation procedure or expert translation panel was used.

### 2.6. Data Management

Audio recordings were transcribed verbatim within 48 h by a professional bilingual transcriptionist bound by a confidentiality agreement, in the language in which each interview had been conducted. The transcriptionist had no role in coding or interpretation. The author checked every transcript against its recording for accuracy. Identifying information was removed and replaced with sequential codes (P1–P17). Audio files were encrypted (AES-256) and stored on a password-protected institutional server; they were deleted after the author had verified the transcripts and completed familiarisation with the recordings (Section 2.7, Phase 1). De-identified transcripts and analytic files are retained in accordance with King Saud University’s data retention policy.

### 2.7. Data Analysis

Analysis followed Braun and Clarke’s six-phase reflexive thematic analysis [37,38,39] and was conducted by the author in NVivo 14 (Lumivero, Denver, CO, USA). Coding was inductive: the theoretical frameworks and the domains of the interview guide were not used as a coding frame, and the frameworks were brought to the analysis only when interpreting the developed themes.

Phase 1: Familiarisation. The author read each transcript at least twice, re-listened to the recordings for tonal and emphatic nuance, and recorded initial observations in reflexive memos.

Phase 2: Coding. Coding generated 168 initial codes across the dataset. Codes were both semantic (e.g., “verbal insult from patient”, “no follow-up after incident”) and latent (e.g., “normalisation of abuse”, “institutional betrayal”). Consistent with reflexive thematic analysis, no second coder was used and no coding reliability coefficient was calculated, since the approach treats the researcher’s interpretive engagement as a resource rather than a source of bias to be controlled [46]. Interpretive breadth was pursued instead through reflexive memoing, deliberate search for accounts that did not fit the emerging framework, and peer debriefing with an external qualitative researcher.

Phase 3: Generating initial themes. Codes were collated into candidate themes and subthemes through iterative mapping, in which the author constructed visual thematic maps, grouped related codes, identified hierarchical relationships, and interrogated provisional themes for coherence. For example, the codes “no follow-up after incident” and “institutional betrayal” were grouped into the candidate subtheme later named absent post-incident response, which was combined with subthemes on policy, training, and staffing to form Theme 3; the codes “verbal insult from patient” and “normalisation of abuse” contributed to the subtheme on verbal aggression within Theme 1.

Phase 4: Reviewing themes. Candidate themes were reviewed at two levels: first against the coded extracts, to confirm that each theme was adequately supported; and then against the full dataset, to confirm that the thematic map represented the overall account. Two candidate themes were merged and one was re-specified during this phase. Participants’ recommendations for improvement were initially organised as a fifth theme. On review, this material was re-specified as a descriptive summary, because it collected participants’ proposed solutions in response to a direct interview question rather than capturing a pattern of shared meaning about their experience; it is reported separately in Section 3.7. The progression from initial codes to final themes, with the participants quoted for each subtheme, is documented in Table S1.

Phase 5: Defining and naming themes. Each theme and subtheme was given a concise, analytically informative name and a written definition specifying its scope, boundaries, and relationship to adjacent themes.

Phase 6: Writing the report. The analytic narrative was constructed, integrating data extracts into an interpretive account addressing the study objectives while maintaining fidelity to participants’ accounts.

### 2.8. Trustworthiness

Rigour was addressed through strategies aligned with Lincoln and Guba’s criteria [47] and with guidance on applying those criteria within thematic analysis [25]. Because data collection and analysis were undertaken by a single researcher, these strategies were selected to bring external and participant perspectives to bear on the analysis at several points.

- Credibility: prolonged engagement with the data across a three-month data collection period; deliberate search during coding for accounts that did not fit the emerging thematic framework; and participant feedback, in which eight participants (8 of 17) reviewed a summary of preliminary findings and reported that the findings were recognisable. Full transcripts were not returned to participants.
- Dependability: an audit trail recording coding decisions, code merges, and changes to candidate themes, which documents the progression from 168 initial codes to the final themes summarised in Table S1; a reflexive journal whose entries were reread at the start of each analytic session; and peer debriefing with an external qualitative researcher who had no involvement in the study and no clinical nursing background. The debriefer reviewed the coding framework, thematic structure, and representative extracts, and was asked to argue against the emerging interpretations rather than to confirm them. Independent double coding was not undertaken (Section 2.7).
- Confirmability: a reflexive journal maintained throughout the study, documenting assumptions, reactions, and evolving interpretations; and traceability of analytic decisions through the audit trail.
- Transferability: description of the research context, participant characteristics, and institutional settings (Section 2.2; Table 1 and Table 2), to allow readers to judge applicability to their own settings.

### 2.9. Researcher Reflexivity

The author is a male nurse academic with clinical experience in medical–surgical nursing and 15 years of combined clinical and research experience. He was a professional insider, sharing participants’ occupational background, but an organisational outsider, with no employment or supervisory role at any participating hospital. Professional insider status supported rapport and contextual understanding during interviews, but also carried risks of unexamined assumption and over-identification with participants’ accounts. The author’s gender may also have affected what female participants were willing to disclose, particularly about sexual harassment. To manage these risks, the author maintained a reflexive journal from the outset of the study, documenting assumptions, emotional reactions, and evolving interpretations, and used scheduled peer debriefing sessions with an external qualitative researcher to test emerging interpretations against an outside reading. Accounts that did not fit the emerging framework were deliberately sought during coding rather than set aside. The author acknowledges that a professional identity as a nurse may have oriented the analysis toward clinician-centred readings, and sought throughout to foreground participants’ own language while engaging critically with institutional and systemic dimensions. Two interpretations were sharpened in response to challenges raised during peer debriefing: reading the absence of post-incident response as communicating to nurses how they were valued (Section 3.5.2), and framing coping as compensation for limited organisational support rather than as an achievement of resilience (Section 4.2). A fuller reflexivity statement is provided as File S3.

### 2.10. Ethical Considerations

The study was approved by the Institutional Review Board of King Saud University, Saudi Arabia (reference KSU-HE-25-1438; approved 15 December 2025). All procedures complied with the Declaration of Helsinki and with Saudi national research ethics regulations.

Written informed consent was obtained from all participants in their preferred language (Arabic or English) before data collection. Consent documentation explained the study aims, procedures, confidentiality protections, audio-recording, the right to withdraw at any time without consequence, and contact details for the author and the ethics committee. Given the sensitivity of the subject matter, each participant was offered referral to occupational health counselling at the end of the interview, irrespective of expressed distress. No adverse events were reported.

## 3. Results

### 3.1. Participant Characteristics

Seventeen nurses participated (Table 2): ten from the two general hospitals and seven from the specialised hospital. The sample comprised 12 women (70.6%) and 5 men (29.4%), with a mean age of 38.4 years (SD 9.2; range 26–55). Eleven participants (64.7%) held a Bachelor of Science in Nursing and six (35.3%) held a nursing diploma. Mean total nursing experience was 11.3 years (SD 8.1; range 2–27), and the mean duration of exposure to WPV was 6.4 years (SD 5.2; range 1–19). Participants worked in emergency departments (*n* = 5), intensive care units (*n* = 4), internal medicine wards (*n* = 5), and other specialty areas (*n* = 3).

Counts reported throughout this section describe the distribution of accounts within this purposive sample of 17 nurses. They indicate how accounts were distributed across participants, not how frequently or severely violence occurred; they are not prevalence estimates and should not be read as generalisable to the wider Saudi nursing workforce. Counts are reported to show how widely a pattern was shared and, where fewer than all participants described it, to indicate variation; accounts that departed from the dominant pattern are noted within the relevant subtheme.

### 3.2. Overview of Themes

**Figure 1:**
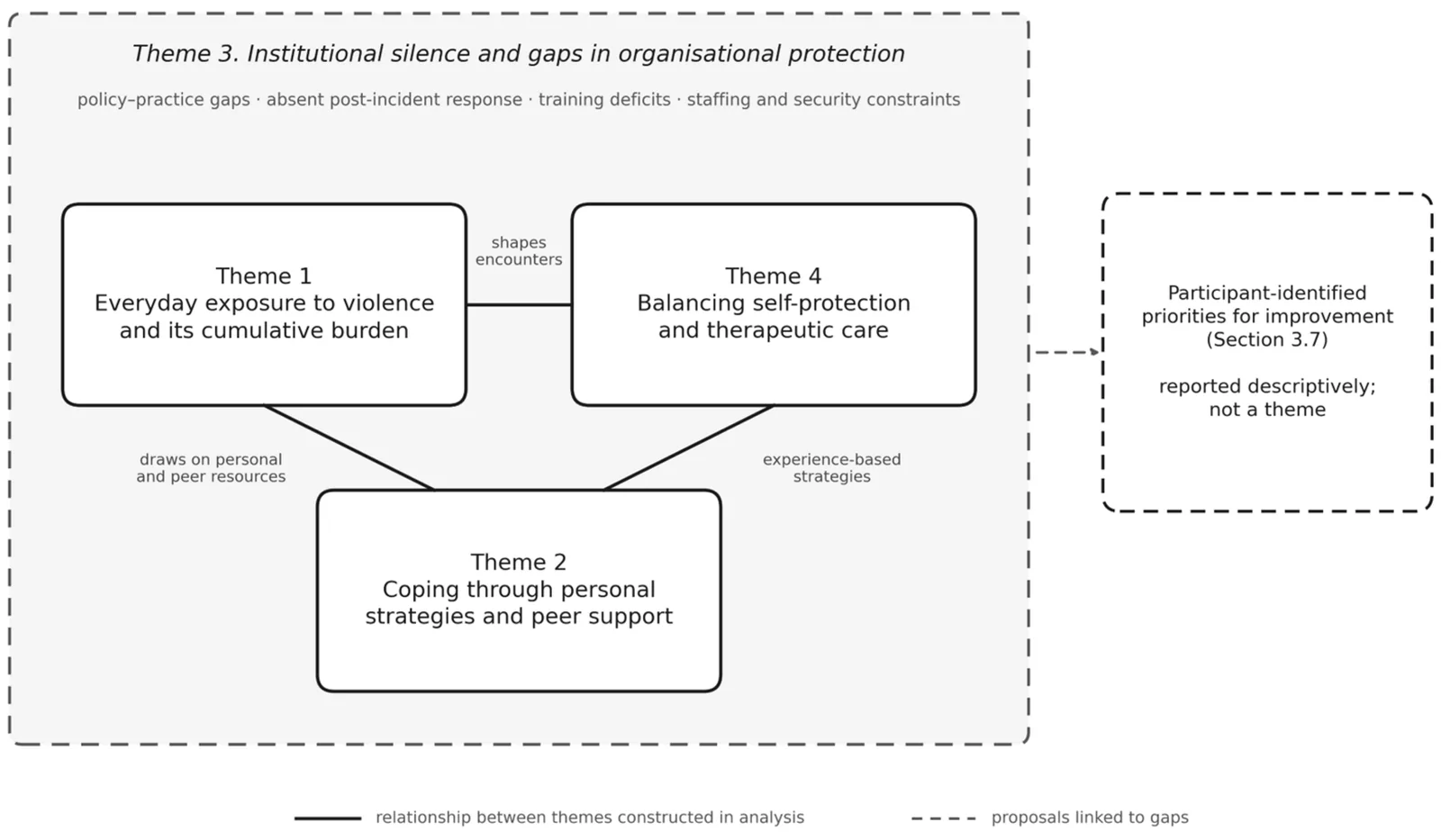
Thematic map of nurses’ experiences of workplace violence. Theme 3 (institutional silence and gaps in organisational protection) frames the setting in which participants described Themes 1, 2, and 4. Lines show relationships between themes as constructed during analysis: in participants’ accounts, exposure to violence (Theme 1) was linked to personal and peer coping (Theme 2) and to how they approached aggressive patients and families (Theme 4), and strategies developed through experience connected Themes 2 and 4. Participant-identified priorities (Section 3.7) are shown outside the thematic structure because they summarise proposed solutions rather than a theme. The map does not depict tested causal or statistical relationships.

Four themes and 13 subthemes were constructed (Table 3; Figure 1). Participants’ priorities for improvement (objective d) are reported separately in Section 3.7 as a descriptive summary rather than as a theme. Quotations are identified by participant code (P1–P17), and every participant is quoted at least once. Table S1 presents the codes contributing to each subtheme and the participants quoted for it.

### 3.3. Theme 1: Everyday Exposure to Violence and Its Cumulative Burden

#### 3.3.1. Verbal Aggression as a Daily Reality

All 17 participants reported verbal aggression, describing it as a routine part of their clinical work. Their accounts included insults directed at their professional competence, nationality, and gender, often from patients and family members. Several participants explained that verbal abuse had become so common that they no longer always recognised it as a form of violence:

“Almost every day, someone raises their voice at us or insults us. Family members can stand right in front of you and tell you that you’re worthless. It happens so often that sometimes we don’t even think of it as violence anymore.”

(P7)

“Patients shout at us and question whether we even know what we’re doing. They say things like, ‘You haven’t learned anything.’ You try to ignore it, but honestly, it affects you.”

(P3)

Participants connected not recognising verbal abuse as violence with rarely reporting it, and described such abuse as largely invisible to organisational reporting and response systems.

#### 3.3.2. Physical Assault and Its Aftermath

Eleven of the 17 participants described experiencing direct physical assault, including being hit, pushed, grabbed, or spat upon. Participants described these incidents as often occurring when patients were agitated, delirious, or intoxicated. They described not only the immediate physical consequences but also an emotional impact that continued afterwards:

“He punched me in the chest and in the face. I fell backwards into the monitor next to the bed. For weeks after that, I was scared whenever I had to deal with an agitated patient. I kept replaying what happened in my head.”

(P5)

“A family member pushed me, and I fell into the medication cart and injured my arm. But what really upset me was that management treated it like it was just a normal part of the job.”

(P11)

Some participants described the way an incident was handled afterwards as adding to its impact, and described institutional dismissal as a further harm; this pattern is developed in Section 3.5.2.

#### 3.3.3. Sexual Harassment and Gendered Vulnerability

Six participants, all of them women (6 of the 12 women in the sample), reported experiences of sexual harassment, including sexually explicit comments, unwanted touching during clinical procedures, and inappropriate advances. Participants described several barriers to reporting, including cultural norms and concerns that their experiences would be dismissed. The remaining six women and the five men did not describe sexual harassment in their interviews; given the sensitivity of the topic and the interviewer’s gender (Section 2.9), this absence cannot be read as an absence of experience:

“He kept making sexual comments and touching my arm in ways that made me uncomfortable. I didn’t report it because I was worried they would just dismiss it.”

(P2)

“Sexual harassment is complicated in our culture. Sometimes people treat these comments as if they’re harmless, but they don’t feel harmless when you’re the one experiencing them. You learn to develop a thick skin, but you really shouldn’t have to.”

(P9)

In these accounts, participants linked their decisions not to report sexual harassment to cultural expectations that such comments are harmless and to expectations that complaints would be dismissed.

#### 3.3.4. Cumulative Psychological Burden

Participants described a range of psychological consequences associated with repeated exposure to WPV, including anxiety, sleep disturbances, intrusive memories, hypervigilance, and emotional exhaustion. Several participants described experiences resembling post-traumatic stress; however, no diagnostic assessment was conducted, and no clinical inference is made:

“When I hear someone raising their voice, my heart starts racing straight away. Sometimes I feel like I’m not the same person I used to be.”

(P8)

“After years of dealing with verbal abuse, physical violence, and harassment, I’m just emotionally exhausted. There have been times when I’ve seriously thought about leaving nursing altogether.”

(P4)

Participants described this burden as accumulating over time rather than arising from isolated incidents, and some, such as P4 and P8, linked it to changes in how they saw themselves or to thoughts of leaving nursing.

### 3.4. Theme 2: Coping Through Personal Strategies and Peer Support

#### 3.4.1. Cognitive and Behavioural Self-Management

All participants described using personal strategies to cope with WPV. These included reframing aggressive behaviour as a response to patient distress, regulating their emotions, exercising, and trying to separate difficult experiences from their personal lives. Although participants viewed these strategies as helpful, they also recognised that personal coping had its limits:

“I try to remind myself that when patients are aggressive, they’re often scared or in pain. When I look at it that way, it doesn’t hurt as much.”

(P1)

“We shouldn’t be expected to deal with all of this on our own by just trying to cope. The hospital has a responsibility to keep us safe too.”

(P6)

The two accounts above illustrate a recurring tension. Some participants, such as P1, described reframing as protective, whereas others, such as P6, rejected the expectation that nurses should cope alone and placed responsibility for preventing and responding to WPV on the organisation.

#### 3.4.2. Peer Solidarity and Informal Debriefing

Fourteen of the 17 participants described their colleagues as their most important source of support; the remaining three did not identify colleagues in this way. Peer relationships provided emotional reassurance, validation, and practical guidance following difficult or violent incidents:

“My colleagues are honestly my lifeline. After that incident, they gathered around me and let me talk about how scared I was. They didn’t make me feel like I was overreacting or try to minimise what happened.”

(P7)

“After difficult incidents, we usually talk to each other. It’s not formal counselling or anything like that, but it’s support from people who really understand what you’ve been through.”

(P12)

Participants described this support as informal and as depending on relationships between colleagues and the culture of individual units rather than on structured organisational support.

#### 3.4.3. Limited Uptake of Formal Support Resources

Although participants described employee assistance programmes and occupational health services as available in principle, they reported limited use of these resources. They described concerns about stigma, difficulties accessing support during working hours, and doubts about whether the available services were appropriate to their needs:

“Technically, counselling is available, but you have to take time away from work, and people know where you’re going. There’s still a lot of stigma, and people worry that others will see them as weak.”

(P10)

“What we really need is support straight after an incident. You shouldn’t have to go home and try to arrange counselling on your own time.”

(P13)

Participants described a gap between the formal availability of support and their ability to access it in practice. In their accounts, support existed on paper but did not feel readily available when they needed it.

### 3.5. Theme 3: Institutional Silence and Gaps in Organisational Protection

#### 3.5.1. Policy–Practice Disconnects

Thirteen of the 17 participants reported that formal violence-prevention policies existed in their hospitals. However, they described these policies as inconsistently communicated, poorly understood, and rarely enforced:

“We have policies about reporting violence, but honestly, I’m not sure I fully understand how the process works. It’s not very clear, and when you’re unsure what to do, you’re less likely to report it.”

(P4)

“Having a policy isn’t enough. If you report something and nothing happens afterwards, then what is the point of the policy?”

(P16)

Participants described a gap between the existence of formal policies and what happened in everyday practice. In their accounts, policies were not always translated into clear procedures or meaningful action following incidents.

#### 3.5.2. Absent Post-Incident Response

The most emotionally charged accounts concerned what happened after violent incidents. Thirteen of the 17 participants reported receiving no follow-up, investigation, or offer of support after experiencing a violent incident:

“After I was assaulted, nobody from management even asked if I was okay. There was no investigation and no consequences. To be honest, being ignored afterwards almost hurt more than the assault itself.”

(P2)

“It makes you feel like they don’t really care about your wellbeing.”

(P11)

Participants often interpreted this lack of response as an indication of how their safety and well-being were valued within the organisation. Some described the absence of support as adding to the impact of the original incident.

The overlap between the two subthemes was complete: the 13 participants who described gaps in post-incident response were the same 13 who reported that formal violence-prevention policies existed in their hospitals. These participants described policies that existed but that, in their experience, were not acted on after incidents. The remaining four participants described neither.

#### 3.5.3. Training Deficits

Eleven of the 17 participants reported that they had never received formal training in de-escalation, conflict resolution, or WPV prevention; the other six had received formal training in at least one of these areas:

“Nobody ever trained me to recognise when someone’s behaviour was escalating, how to de-escalate the situation, or even how to protect myself. Everything I’ve learned has been through experience.”

(P5)

“It would really help to have training on recognising psychiatric symptoms and using de-escalation techniques. We’re expected to handle very complicated situations, but sometimes we have very little preparation.”

(P17)

Participants described learning largely through experience and emphasised the need for more structured preparation to help them recognise and respond to potentially violent situations.

#### 3.5.4. Staffing and Infrastructure Constraints

Participants identified understaffing, limited security presence during high-risk periods, and the absence of emergency communication devices as structural factors that they perceived as increasing their vulnerability:

“When something happens, there often aren’t enough staff around to help. One nurse might be trying to calm an aggressive patient while still looking after everyone else.”

(P6)

“Having panic buttons would make a huge difference. Just knowing that you could call for help immediately would make you feel much safer.”

(P9)

Participants viewed these limitations as organisational issues and associated them with difficulty responding safely when incidents occurred.

### 3.6. Theme 4: Balancing Self-Protection and Therapeutic Care

#### 3.6.1. Communication Barriers with Agitated Patients

Participants described the challenges of managing aggressive patients in real time and associated these challenges with linguistic diversity, cognitive impairment, and psychiatric symptoms:

“When a patient becomes aggressive, I’m trying to work out what’s upsetting them while also thinking about my own safety. Sometimes they don’t speak Arabic or English very well, and that makes everything even harder.”

(P7)

“I think understanding more about patient psychology would help. If I understood better why someone was becoming aggressive, I might be able to respond in a better way.”

(P12)

Participants described the need to make rapid decisions while simultaneously trying to understand the causes of a patient’s behaviour and maintain their own safety.

#### 3.6.2. Maintaining Compassion While Setting Boundaries

A recurring tension involved balancing personal safety with the professional expectation to provide compassionate and patient-centred care:

“Setting boundaries with a hostile patient isn’t easy. I need to protect myself, but at the same time, I’m still expected to be compassionate and provide proper care.”

(P8)

“Even when patients are being hostile, I try to remind myself that they might be scared or in pain. It’s difficult, but I still try to stay compassionate.”

(P3)

Participants described learning to manage this tension over time. Their accounts suggested that they developed practical ways of adjusting their communication and approach as situations became more threatening while still attempting to maintain therapeutic engagement. Participants attributed this knowledge largely to experience rather than to formal training or institutional guidance, consistent with the training gaps described in Section 3.5.3.

### 3.7. Participant-Identified Priorities for Improvement

This section addresses objective (d). It summarises the improvements participants proposed when asked what would reduce violence (File S2, domain v). The proposals are presented descriptively as participants’ views; they were not evaluated in this study. Implications formulated by the author, which draw on these proposals and on published evidence, are presented separately in Section 4.9.

#### 3.7.1. Mandatory Education and Skill Development

All participants supported mandatory education as a way to improve workplace safety. They emphasised the need for practical training in recognising escalation, using de-escalation techniques, and communicating safely with distressed or aggressive patients:

“Every nurse should have mandatory training in de-escalation. We need to know how to recognise when things are escalating, how to communicate calmly, and how to use non-threatening body language. I think that could prevent a lot of violence.”

(P1)

“We need to understand psychiatric symptoms, why some patients become aggressive, and what de-escalation actually looks like in practice.”

(P14)

Participants suggested that this training should be provided regularly, assessed for competency, and incorporated into orientation and continuing professional education rather than being offered only as an optional activity.

#### 3.7.2. Policy Reform and Safety-Culture Change

Participants described the need for broader organisational change. Their recommendations included clear zero-tolerance policies, easier reporting systems, transparent investigations, visible accountability, and timely support following violent incidents:

“The culture needs to change. Instead of asking, ‘What did the nurse do to provoke this?’, we should be asking, ‘What can we do to prevent this from happening again?’”

(P6)

“A real safety culture means that leaders don’t just talk about preventing violence. They actually invest in it and provide the resources we need.”

(P15)

Participants linked these recommendations to their experiences of gaps within their own workplaces. They identified measures such as panic buttons, environmental changes to reduce isolation, stronger security, adequate staffing, and visible leadership accountability as interconnected components of a broader strategy to improve nurse safety. Several proposals, such as panic buttons and immediate post-incident support, restated gaps already described in Theme 3, which is why they are reported here as priorities rather than as a separate theme.

## 4. Discussion

This study describes how nurses in three Riyadh hospitals experienced, managed, and interpreted WPV. Four themes were constructed. Taken together, participants’ accounts describe personal and peer coping carrying much of the burden of responding to violence in settings where participants perceived institutional protection as inadequate. In the sections that follow, what participants reported is distinguished from the author’s interpretation, which draws on stress and coping theory [26] and COR theory [27,28].

### 4.1. Violence as a Routine and Cumulative Exposure

Participants did not describe violence as a series of exceptional events. They described it as a routine part of their work, with verbal aggression at the centre; several said they no longer always recognised it as violence. Normalisation and under-reporting of WPV have been documented in other settings [2,30,48], including a qualitative study of psychiatric nurses in Turkey in which most participants regarded violence as a normal part of their work [49], and Saudi qualitative studies in a government hospital [33] and in inpatient mental health facilities [34] have likewise described violence as a recurring feature of nurses’ working lives. The present data cannot show whether normalisation is stronger in Saudi hospitals than elsewhere. Participants themselves linked it to the frequency of abuse (P7) and to managers treating violence as part of the job (P11). Cultural norms around communication and family involvement in care may also contribute, but this is an interpretation that the study was not designed to test. A read through of COR theory [27] indicates normalised verbal aggression can be interpreted as an unrecorded resource loss: participants described it as rarely reported, yet as wearing down their emotional reserves (P4, P8).

Accounts of physical assault and sexual harassment in this sample cannot be compared with survey prevalence figures; what they add is qualitative. Two of the women who described sexual harassment explained why it went unreported or unchallenged: P2 expected to be dismissed, and P9 described such comments being treated as harmless. A systematic review has documented sexual harassment of female nurses across countries [50], and a Saudi national survey found sexual violence to be more common among women, nurses, and night-shift workers [19]. P9 explicitly located the difficulty in cultural expectations. Whether such expectations make disclosure harder in Saudi hospitals than in other settings cannot be determined from these data, but, in the author’s interpretation, reporting systems that rely on the affected nurse to initiate a formal complaint may be poorly suited to this form of violence; this was not examined directly.

### 4.2. Coping as Compensation for Limited Organisational Support

Participants described coping strategies that, in Lazarus and Folkman’s terms [26], combined emotion-focused efforts, such as reappraising aggression as a sign of patient fear or pain (P1), with seeking social support from colleagues. What distinguished their accounts was less the strategies themselves than the conditions under which they were used: participants connected their reliance on these strategies with formal support that they described as absent, difficult to reach during shifts, or stigmatised (Section 3.4.3). COR theory [27,28] offers one reading of this pattern: it proposes that people facing resource loss invest their remaining personal and social resources to limit further loss and that sustained loss can deplete those reserves. Participants did not uniformly accept this arrangement; P6 stated that nurses “shouldn’t be expected to deal with all of this on our own”. The language of resilience therefore needs care. In the author’s view, recognising nurses’ capacity to cope is warranted, but treating the absorption of harm as a professional virtue may obscure what participants described as insufficient organisational protection.

Participants’ reliance on peer support is consistent with international qualitative evidence on the importance of informal support from colleagues after violent incidents [49] and on nurses’ unmet support needs following violence [29]. In this sample, however, peer support depended on relationships within individual units and was not organised by the hospitals. Structured peer-support programmes of the kind implemented for healthcare staff affected by adverse events [51] could build on this existing resource and make it available more consistently across units, although their effectiveness after WPV would need to be evaluated.

### 4.3. Institutional Silence After Incidents

Participants did not describe the absence of follow-up, investigation, or support after violent incidents only as a missing service. P2 said that being ignored afterwards “almost hurt more than the assault itself”, and P11 read the absence of response as a sign that management did not care about nurses’ well-being. Organisational justice research, which links employees’ perceptions of fair treatment by their organisation to their trust, commitment, and well-being [52], offers one interpretive lens: from this perspective, participants’ accounts of being ignored after an incident can be read as experiences of unfair treatment by their organisation. Smith and Freyd [53] define institutional betrayal as wrongdoing by an institution against individuals who depend on it, including failure to respond supportively when harm occurs. Applying this concept to the present data is the author’s interpretation; participants did not use the term, although the latent code “institutional betrayal” was generated during analysis (Section 2.7).

In the present sample, the participants who knew that policies existed were the same participants who described the absence of response after incidents (Section 3.5.2). P16 described reporting as pointless when nothing happened afterwards, which suggests that policies that are not visibly acted upon may discourage reporting. Australian emergency nurses in a Delphi study similarly agreed that enforcing zero-tolerance policy, with managers following up reports of violence, would improve staff safety [54], and reviews of nurses’ reporting behaviour [30] and post-incident support needs [29] address related concerns. These readings rest on participants’ reports and were not tested; the study did not independently assess policy content, reporting data, or organisational performance.

### 4.4. Balancing Self-Protection and Therapeutic Care

Participants described managing aggressive encounters as a double task: working out why a patient or relative was escalating while protecting themselves, often with limited shared language (P7). A similar conflict has been described among nurses on acute psychiatric inpatient units, who at times chose self-protection over patient care when they felt afraid or lacked confidence in managing violence [22]. Participants in the present study described trying to hold both commitments, setting boundaries while remaining compassionate (P3, P8), and learning to do so through experience rather than training. Language and cultural differences between nurses and patients are a documented barrier to nurse–patient communication in Saudi Arabia [15], and P7 described limited shared language as making aggressive encounters harder to manage. Because nationality data were not collected (Section 4.8), the study cannot show how these difficulties differed between Saudi and expatriate nurses. Because participants described trying to sustain therapeutic engagement while feeling unsafe and unprepared, the consequences of violence may extend to patient care; this possibility was not examined directly.

### 4.5. Participant-Identified Priorities: Training as Necessary but Insufficient

Participants identified the lack of formal training in de-escalation, conflict resolution, or violence prevention (Section 3.5.3) as a priority. The intervention evidence supports a qualified reading of training as a solution. A systematic review of 26 intervention studies grouped interventions into standalone training, structured education programmes, and multicomponent interventions that added organisational changes such as reporting systems and safety procedures; training featured in almost all of them [55]. A systematic review of 15 emergency department (ED) intervention studies, 11 of which evaluated training programmes, found that most reported positive effects on violent incidents or staff preparedness, but the small number and limited methodological quality of the studies meant that these findings required confirmation [56]. Reviews of ED-specific approaches describe training alongside environmental and organisational measures [57,58], and a systematic review of five studies of de-escalation training in forensic psychiatric settings found no relevant effect on the rate of violent incidents, although staff reported feeling safer [59]. Guidance on developing and evaluating complex interventions [60] is also relevant, because programmes combining education, environmental modification, policy reform, and cultural change need to be designed and evaluated as interacting components; participants proposed a similar combination of measures (Section 3.7).

### 4.6. Integrative Interpretation: Informal Coping Within Limited Institutional Protection

Read together, the themes describe a connected pattern rather than four separate findings. Exposure to violence was frequent and, for verbal aggression, largely invisible to organisational systems (Theme 1). Participants responded by drawing on personal strategies and colleagues (Theme 2). Participants connected this reliance with organisational resources they described as missing or difficult to reach: policies were not acted on, incidents went without follow-up, most participants had not been trained, and staffing and security were limited (Theme 3). Participants described the same gaps in the clinical encounter, where they tried to remain therapeutically engaged with aggressive patients and families while relying on experience rather than preparation (Theme 4). A read through of COR theory [27,28] indicates this pattern can be interpreted as the work of protection shifting from the organisation to individuals and peer networks, with participants describing its costs in cumulative distress and in the strain of maintaining therapeutic care. This synthesis is the author’s interpretation of relationships between themes; a single-timepoint qualitative study cannot establish that institutional gaps caused the patterns described. It also suggests, cautiously, that interventions focused only on individual resilience would not address the organisational gaps participants identified.

### 4.7. Contextual Contributions

This study makes three contributions to the Saudi Arabian and wider Gulf literature. First, it examines nurses’ WPV experiences across hospitals differing in ownership and specialisation within a single city, extending earlier Saudi qualitative work conducted in a single government hospital [33] and in inpatient mental health facilities [34], and responding to the evidence gap identified in a national narrative review [35]. Second, it reports dynamics that participants linked to their social and cultural context, particularly around sexual harassment disclosure and family involvement in violent episodes, which survey instruments are not designed to capture. Third, it describes how participants connected the absence of post-incident response with their reliance on informal coping. The improvement priorities reported reflect frontline knowledge, although their feasibility and effectiveness would need to be evaluated prospectively.

### 4.8. Strengths and Limitations

The study has several methodological strengths. The qualitative descriptive design supported detailed exploration of a complex and sensitive phenomenon. Recruitment from three hospitals differing in ownership and specialisation widened contextual range. Trustworthiness strategies included participant feedback on preliminary findings, deliberate search for disconfirming accounts, an audit trail, reflexive journaling, and peer debriefing with an external qualitative researcher. Reporting follows COREQ, and the analytic audit trail from codes to themes is provided as Supplementary Materials.

Several limitations warrant consideration. Data were collected at a single timepoint and rely on participants’ retrospective accounts; prospective or longitudinal qualitative designs would be better suited to examining how experience and coping evolve. Purposive sampling from three Riyadh hospitals provided variation in ownership and specialisation, but the findings are not intended to transfer directly to rural settings, primary care, or healthcare systems outside the Gulf region, and readers should assess transferability against the contextual description provided. Participant characteristics were reported by facility category rather than by hospital and unit, which limits readers’ ability to judge how accounts varied across sites. With five men among 17 participants, male nurses’ experiences may be underrepresented. The interviewer was male, and female participants may have been less willing to describe sexual harassment in detail; the counts for sexual harassment should be read with this in mind. The demographic data reported do not include participant nationality, so the interaction between workforce composition and violence experience, identified in the Introduction as a feature of the Saudi context, could not be examined. The accounts reported here concern mainly violence from patients and their relatives; violence between staff, which Saudi qualitative work has also described [33], was not examined separately. Recruitment through nurse managers and snowball referral, despite safeguards against coercion, may have introduced selection bias, particularly if nurses fearing retribution declined to participate; this possibility cannot be empirically assessed. Reliance on retrospective self-report is subject to recall and social desirability bias, which in-depth interviewing with probing does not remove. Finally, ten of the seventeen interviews were conducted in Arabic and the quotations reproduced here were translated into English by the researcher without a formal forward and back-translation procedure or independent verification. Analysis was conducted on transcripts in their original language, which was intended to preserve meaning at the analytic stage, but readers should note that the English quotations represent one translator’s rendering and that some linguistic nuance is inevitably lost (see Section 2.5).

Data collection, coding, and theme development were undertaken by a single researcher, and no independent coder reviewed the transcripts or codes. Reflexive thematic analysis does not require multiple coders or inter-coder agreement, because themes are understood as the product of the researcher’s interpretive engagement rather than as entities to be reliably detected [39,46]. This does not remove the risk of researcher bias. The author’s professional identity as a nurse, his gender, and his assumptions about institutional responsibility could have shaped which topics were probed, which codes were generated, and which patterns were foregrounded. Professional transcription, reflexive journaling, deliberate attention to accounts that did not fit, peer debriefing with an external qualitative researcher, and participant feedback on preliminary findings were intended to address this risk but cannot eliminate it. Participant feedback was obtained from eight participants on a summary of preliminary findings rather than on full transcripts or the final thematic structure. Readers should weigh the findings with these constraints in mind.

### 4.9. Implications for Practice, Education, Management, Policy, and Research

The implications in this section are the author’s, drawing on participants’ proposals (Section 3.7) and the cited intervention evidence. They point to measures that hospitals could consider and evaluate. At the organisational level, these include recurrent, competency-assessed de-escalation and violence-prevention training for clinical staff; reporting systems that are simple to use, lead to visible investigation, and give feedback to the reporting nurse; post-incident protocols that include prompt supervisory contact, access to occupational health support during working hours, and structured follow-up; and safety infrastructure such as personal alarms and security presence during high-risk periods. Participants described these components as dependent on one another, and intervention reviews include programmes that combine training with organisational change [55,56]; such programmes should be designed and evaluated using methods suited to complex interventions [60]. Their effectiveness in Saudi hospitals has not yet been established.

For nursing education, the findings suggest that pre-registration programmes and hospital orientation could include recognition of escalation, de-escalation, communication with distressed families, and communication across language and cultural differences, together with guidance on reporting and on seeking support after an incident. At the policy level, a study of 17 nurses in three hospitals cannot support specific national recommendations; the patterns described here could instead inform the questions examined in larger, multi-site studies of reporting and post-incident response.

At the research level, intervention studies evaluating the effectiveness and implementation of multicomponent WPV-prevention programmes in Gulf healthcare settings are needed, as are longitudinal qualitative studies tracking the temporal dynamics of violence exposure, coping, and recovery. Future studies should also record nurses’ nationality, report findings by site where confidentiality allows, and include the perspectives of patients, families, and nurse managers.

## 5. Conclusions

Nurses in three Riyadh hospitals described WPV as frequent and varied, with verbal aggression so routine that it was often not recognised or reported. They managed its effects mainly through personal coping and support from colleagues, and they described doing so in organisations where most knew that violence-prevention policies existed but post-incident follow-up, training, staffing, and security were, in their experience, inadequate. Participants described the same gaps as making it harder to remain therapeutically engaged with aggressive patients and families. In participants’ accounts, individual resilience was not a sufficient response to WPV. The organisational arrangements they identified, namely reporting, post-incident follow-up, training, staffing, and security, are the areas the findings point to for further attention and evaluation. Because the findings come from 17 nurses in three hospitals in one city, their transferability to other settings should be judged against the contextual description provided (Section 4.8).

## Figures and Tables

**Table 1:** Characteristics of the participating hospitals.

Characteristic	Hospital A	Hospital B	Hospital C
Ownership	Government	Government	Private
Service profile	Tertiary regional referral centre	Specialised (psychiatric, oncology, rehabilitation)	Multi-specialty
Bed capacity	>500	>500	>500
Reporting category (Table 2)	General	Specialised	General
Participants, n	10 (A and C combined)	7	Included with A

Participant numbers are reported together for Hospitals A and C, the two general hospitals, consistent with the facility categories used in Table 2; clinical units for the whole sample are reported in Table 2.

**Table 2:** Participant characteristics (*n* = 17).

Characteristic	*n* (%) or Mean ± SD
Age (years)	38.4 ± 9.2 (range 26–55)
Gender	
Female	12 (70.6)
Male	5 (29.4)
Educational qualification	
BSc Nursing	11 (64.7)
Diploma in nursing	6 (35.3)
Total nursing experience (years)	11.3 ± 8.1 (range 2–27)
Duration of exposure to WPV (years)	6.4 ± 5.2 (range 1–19)
Department	
Emergency	5 (29.4)
Intensive care unit	4 (23.5)
Internal medicine	5 (29.4)
Other	3 (17.6)
Facility type	
General hospital	10 (58.8)
Specialised hospital	7 (41.2)

WPV = workplace violence; BSc = Bachelor of Science; SD = standard deviation. Facility categories are defined in Section 2.2 and Table 1. Percentages are rounded to one decimal place and may not sum to exactly 100. Duration of exposure to WPV was self-reported.

**Table 3:** Themes and subthemes.

Theme	Subthemes
1. Everyday exposure to violence and its cumulative burden	1.1 Verbal aggression as a daily reality; 1.2 Physical assault and its aftermath; 1.3 Sexual harassment and gendered vulnerability; 1.4 Cumulative psychological burden
2. Coping through personal strategies and peer support	2.1 Cognitive and behavioural self-management; 2.2 Peer solidarity and informal debriefing; 2.3 Limited uptake of formal support resources
3. Institutional silence and gaps in organisational protection	3.1 Policy–practice disconnects; 3.2 Absent post-incident response; 3.3 Training deficits; 3.4 Staffing and infrastructure constraints
4. Balancing self-protection and therapeutic care	4.1 Communication barriers with agitated patients; 4.2 Maintaining compassion while setting boundaries

## Data Availability

The de-identified datasets collected and analysed during this study are not publicly available in order to protect participant confidentiality but are available from the corresponding author on reasonable request and subject to ethical approval.

## Supplementary Materials

The following supporting information can be downloaded at https://www.mdpi.com/article/10.3390/healthcare14193341/s1: File S1: COREQ 32-item checklist; File S2: Semi-structured interview guide; Table S1: Code-to-theme progression framework, with contributing participants; File S3: Researcher reflexivity statement.

## Abbreviations

The following abbreviations are used in this manuscript:

COREQ	Consolidated Criteria for Reporting Qualitative Research
ED	Emergency department
WPV	Workplace violence
