# Supplementary material for: Institutional Silence and Informal Coping: A Qualitative Descriptive Study of Nurses’ Experiences of Workplace Violence in Three Hospitals in Riyadh, Saudi Arabia
Source: Healthcare (Basel). 2026 Oct 7;14(19):3341. doi: 10.3390/healthcare14193341 (PMC13664904; doi:10.3390/healthcare14193341)
Supplement: Supplementary file 1 [file healthcare-14-03341-s001.zip › healthcare-4557181-supplementary.pdf]

Supplementary Files (Supplementary Materials)

File S1. COREQ 32-Item Checklist

Consolidated Criteria for Reporting Qualitative Research (Tong, A.; Sainsbury, P.; Craig, J. *Int. J. Qual. Health Care* 2007, 19, 349–357). Section numbers refer to the submitted manuscript.

Domain 1: Research team and reflexivity

Personal characteristics

No.	Item	Guide question	Reported in
1	Interviewer/facilitator	Which author conducted the interviews?	Section 2.4. All 17 interviews were conducted by the sole author (A.M.A.).
2	Credentials	What were the researcher's credentials?	Sections 2.4 and 2.9. Doctoral qualification in nursing.
3	Occupation	What was their occupation at the time of the study?	Section 2.9. Nurse academic, Department of Medical-Surgical Nursing, King Saud University.
4	Gender	Was the researcher male or female?	Section 2.4. Male.
5	Experience and training	What experience or training did the researcher have?	Sections 2.4 and 2.9. Formal training in qualitative interviewing; 15 years of combined clinical and research experience; clinical background in medical–surgical nursing.

Relationship with participants

No.	Item	Guide question	Reported in
6	Relationship established	Was a relationship established prior to study commencement?	Section 2.4. No pre-existing clinical or supervisory relationship with any participant; the author was not employed by any participating hospital.
7	Participant knowledge of the interviewer	What did participants know about the researcher?	Sections 2.4 and 2.10, and the guide preamble in File S2. Participants were informed of the author's identity as a nurse academic, the purpose of the study, and its academic (non-managerial) basis during recruitment and the consent process.
8	Interviewer characteristics	What characteristics were reported about the interviewer?	Section 2.9 and File S3. Insider positioning as a nurse, its analytic advantages and risks, and the strategies used to manage them.

Domain 2: Study design

Theoretical framework

No.	Item	Guide question	Reported in
9	Methodological orientation and theory	What methodological orientation underpinned the study?	Section 2.1. Qualitative description with reflexive thematic analysis, under a broadly constructionist epistemological orientation. Lazarus and Folkman's transactional model and Hobfoll's Conservation of Resources theory informed the study (Section 1).

Participant selection

No.	Item	Guide question	Reported in
10	Sampling	How were participants selected?	Section 2.3. Purposive sampling supplemented by snowball referral. Eligibility criteria are listed in full.
11	Method of approach	How were participants approached?	Section 2.3. Three channels: liaison with unit nurse managers, informational posters in staff break rooms, and referral from enrolled participants.
12	Sample size	How many participants were in the study?	Sections 2.3 and 3.1. N = 17.
13	Non-participation	How many refused to participate or dropped out, and why?	Section 2.3. 25 eligible nurses were approached; 8 declined, citing work-schedule and shift-related constraints; 17 consented and were interviewed; no participant withdrew after consenting.

Setting

No.	Item	Guide question	Reported in
14	Setting of data collection	Where were the data collected?	Sections 2.2 and 2.4. Three hospitals in Riyadh (tertiary government, government specialised, private multi-specialty); interviews held in private rooms within or near the participants' hospitals.
15	Presence of non-participants	Was anyone else present?	Section 2.4. No one else was present. No supervisor was present during consent or interview (Section 2.3).
16	Description of sample	What are the important characteristics of the sample?	Section 3.1 and Table 1. Age, gender, qualification, nursing experience, duration of WPV exposure, department, and facility type.

Data collection

No.	Item	Guide question	Reported in
17	Interview guide	Were questions, prompts, and guides provided?	Section 2.4 and File S2. Twenty open-ended questions across five domains, plus a demographic section and standard probes. The guide was piloted with two eligible nurses who were not among the 25 approached, and refined for clarity and flow; their data are not included.
18	Repeat interviews	Were repeat interviews carried out?	Section 2.4. No repeat interviews were conducted. Eight participants subsequently reviewed a written summary of preliminary findings (Section 2.8); this was not a second interview.
19	Audio/visual recording	Was audio or visual recording used?	Section 2.4. All interviews were audio-recorded with explicit consent, using an encrypted digital recorder. No visual recording.
20	Field notes	Were field notes made during and/or after the interview?	Section 2.4. Field notes on contextual observations, non-verbal cues, and the author's reflections were completed immediately after each session.
21	Duration	What was the duration of the interviews?	Section 2.4. 28 to 52 minutes; mean 38 minutes.
22	Data saturation	Was data saturation discussed?	Section 2.3. Saturation is **not** claimed. Sample size was guided by information power (Malterud et al.), and the manuscript notes that the developers of reflexive thematic analysis regard

No.	Item	Guide question	Reported in
			saturation as conceptually incompatible with the approach. It is recorded descriptively that coding of successive transcripts yielded no codes absent from the framework after Interview 15, and that two further interviews (P16, P17) were conducted; this informed but did not determine the decision to conclude recruitment.
23	Transcripts returned	Were transcripts returned to participants for comment or correction?	Section 2.8. No. Full transcripts were not returned. Eight of the 17 participants reviewed a written summary of preliminary findings and confirmed that these were recognisable and resonant (see item 28).

Domain 3: Analysis and findings

Data analysis

No.	Item	Guide question	Reported in
24	Number of data coders	How many data coders coded the data?	Section 2.7. One (the author). Consistent with reflexive thematic analysis, no second coder was used and no coding reliability coefficient was calculated. Interpretive breadth was pursued through reflexive memoing, deliberate search for accounts that did not fit the emerging framework, and peer debriefing with an external qualitative researcher.
25	Description of the coding tree	Was a description of the coding tree provided?	Section 2.7 and Table S1. Progression from 168 initial codes through collapsed codes and candidate themes to 5 final themes and 15 subthemes.
26	Derivation of themes	Were themes identified in advance or derived from the data?	Section 2.7. Coding was primarily inductive; themes were constructed from the data.
27	Software	What software was used to manage the data?	Section 2.7. NVivo 14 (Lumivero, Denver, CO, USA).
28	Participant checking	Did participants provide feedback on the findings?	Section 2.8. Eight of the 17 participants reviewed a written summary of preliminary findings and confirmed that these were recognisable and resonant.

Reporting

No.	Item	Guide question	Reported in
29	Quotations presented	Were participant quotations presented to illustrate the themes?	Sections 3.3 to 3.7. Each subtheme is illustrated by at least two verbatim extracts, identified by de-identified participant codes (P1–P17). Ten interviews were conducted in Arabic; translation of quotations is described in Section 2.5.
30	Data and findings consistent	Was there consistency between the data presented and the findings?	Section 3. Every analytic claim is accompanied by supporting extracts; counts are reported as proportions of this purposive sample and are explicitly not prevalence estimates (Section 3.1).
31	Clarity of major themes	Were major themes clearly presented?	Table 2, Figure 1, and Sections 3.3 to 3.7. Five themes, each defined and illustrated.
32	Clarity of minor themes	Is there a description of diverse cases or minor themes?	Sections 3.3 to 3.7. Fifteen subthemes are reported. Within-theme variation is described at Section 3.4.3 (formal support nominally available but not taken up) and Section 3.6.2

No.	Item	Guide question	Reported in
			(participants differed in how they balanced self-protection against therapeutic engagement). See the note below.

File S2. Semi-Structured Interview Guide

Preamble

Thank you for agreeing to take part. I am a nurse academic at King Saud University, and this interview is for a research study, not for your hospital or your manager. It will take about 30 to 45 minutes. I will ask about your experiences of workplace violence. There are no right or wrong answers, and I am interested in your honest perspective. You can stop at any time, take a break, or skip any question you would prefer not to answer, and nothing you say will be identifiable in anything I publish. May I begin recording?

Section A: Demographic information

1. Age (years)
2. Gender
3. Educational qualification
4. Total years of nursing experience
5. Years of experience of workplace violence
6. Department or unit (emergency / intensive care / internal medicine / other)
7. Type of healthcare facility (general / specialised)

Section B: General experience

1. Can you describe your experience of facing workplace violence?
   - *Probe: Can you give me a specific example?*
2. What types of violence have you encountered or witnessed in the hospital?
   - *Probe: Verbal, physical, sexual, other?*
3. How does violence affect your daily work and the quality of care you give?
4. Have you ever felt unsafe while working?
   - *Probe: What was happening at the time? How did you respond?*

Section C: Violence management strategies

5. What strategies do you use to protect yourself when facing violence?
6. How do you respond to aggressive patients or family members?
7. Do you have ways of handling repeated or potential violent incidents?
8. Do you receive support from colleagues or from the administration when violence occurs?
   - *Probe: What kind of support? Is it adequate?*

Section D: Influencing factors

9. What factors help you manage violence effectively?
10. What factors make it more difficult to deal with violence?
11. How does institutional support affect your sense of safety?
12. Are the available resources sufficient, and how do they affect your work?
13. How do hospital policies influence violence management and staff protection?

Section E: Interaction with patients and families

14. How do you handle aggressive behaviour from patients or families?
15. What challenges do you face when communicating with aggressive patients?
16. How does patient and family behaviour affect your professionalism and your sense of safety?
17. Do you find it difficult to balance providing care with protecting yourself and your team?

Section F: Development and improvement

18. What additional training or support would help you improve your violence management skills?
19. What recommendations would you make to improve the work environment and reduce violence?
20. Do you have suggestions for hospital policies or staff support programmes that would improve workplace safety?

General probes used throughout

- Can you tell me more about that?
- How did that make you feel?
- What happened next?
- Can you give me an example?
- What did you think at the time?

Closing

Thank you for your time and your openness. Is there anything else you would like to add about your experience? Everything you have shared today will remain confidential. If this conversation has raised any concerns for you, I can give you information about the counselling and support services available to you through occupational health.

Table S1. Code-to-Theme Progression Framework

Illustrative progression from initial codes to final themes and subthemes. Theme and subtheme labels match Table 2 of the manuscript exactly.

Illustrative initial codes (Phase 2)	Collapsed codes (Phase 3)	Candidate theme (Phase 3)	Final theme (Phase 5)	Final subtheme (Phase 5)
Shouted at; called names; insulted; hostile comments from family	Forms of verbal aggression	Verbal abuse as routine violence	1. The continuum of violence exposure	1.1 Verbal aggression as a daily reality
Punched; pushed; spat upon; grabbed during care	Physical violence incidents	Physical harm and psychological echo	1. The continuum of violence exposure	1.2 Physical assault and its aftermath
Sexual comments; unwanted touching; inappropriate advances	Sexual harassment forms	Gendered and silenced violence	1. The continuum of violence exposure	1.3 Sexual harassment and gendered vulnerability
Anxiety; insomnia; hypervigilance; intrusive memories; considering leaving nursing	Psychological consequences	Cumulative burden	1. The continuum of violence exposure	1.4 Cumulative psychological burden
Reframing aggression as symptom; breathing exercises; physical exercise	Individual coping repertoire	Personal survival strategies	2. Navigating survival: coping and resilience	2.1 Cognitive and behavioural self-management
Colleague validation; informal debriefing; shared understanding	Peer support practices	Solidarity as resource	2. Navigating survival: coping and resilience	2.2 Peer solidarity and informal debriefing
Stigma of counselling; inaccessible employee assistance; perceived irrelevance	Barriers to formal support	Availability versus accessibility	2. Navigating survival: coping and resilience	2.3 Limited uptake of formal support resources
Policies exist but unclear; not communicated; not enforced	Policy existence without implementation	Institutional double standard	3. Institutional silence and structural gaps	3.1 Policy–practice disconnects
No follow-up; no investigation; no management response	Post-incident organisational silence	Second injury	3. Institutional silence and structural gaps	3.2 Absent post-incident response
Never trained in de-escalation; learned by experience	Training absence	Preparation deficit	3. Institutional silence and structural gaps	3.3 Training deficits
Short-staffed; no security presence; no panic buttons	Resource constraints	Structural vulnerability	3. Institutional silence and structural gaps	3.4 Staffing and infrastructure constraints
Language barriers; confusion; psychiatric symptoms	Communication obstacles	Interactional complexity	4. The relational tightrope: patient–nurse interactions	4.1 Communication barriers with agitated patients
Maintaining compassion; setting boundaries; balancing safety	Therapeutic tension	Professional dual mandate	4. The relational tightrope: patient–nurse interactions	4.2 Balancing safety with therapeutic engagement
Mandatory training; de-escalation; annual refresher	Training recommendations	Education as intervention	5. Charting pathways to safety	5.1 Mandatory education and skill development
Zero tolerance; clear reporting; accountability; culture shift	Policy and culture reform	Systemic transformation	5. Charting pathways to safety	5.2 Policy reform and safety-culture transformation

File S3. Researcher Reflexivity Statement

Positioning

This study was designed, conducted, and analysed by a single researcher: a nurse academic in the Department of Medical-Surgical Nursing at King Saud University, with a clinical background in medical–surgical nursing and 15 years of combined clinical and research experience. The study is therefore an instance of insider research, a nurse investigating nurses' experiences of violence, and that position shaped the work at every stage.

What the position made possible

A shared professional vocabulary allowed rapport to be established quickly and made it unnecessary for participants to explain clinical context. Several participants remarked during interviews that they were speaking more openly because they were addressing another nurse. Familiarity with the organisational structures of Saudi hospitals, including reporting lines, shift patterns, and the role of family members in patient care, made it possible to probe institutional dynamics with specificity rather than generality.

What the position risked

Three risks were identified at the outset.

The first was over-identification: accepting participants' accounts uncritically because they resonated with the author's own clinical experience. The second was professional socialisation, which could predispose the analysis toward clinician-centred readings at the expense of organisational, managerial, or patient perspectives. The third concerned cultural assumptions about the causes of violence and about which responses count as acceptable, particularly in relation to gender, family involvement in care, and the disclosure of sexual harassment.

A fourth consideration arose from the study's design rather than its author's identity. Because coding and theme construction were undertaken by one person, there was no second analyst whose reading might have unsettled the first.

How these were managed

A reflexive journal was kept from the design stage through to the final draft, recording assumptions carried into each interview, emotional reactions during and after interviews, and the way interpretations changed across the analysis. Entries were reread at the start of each analytic session.

Peer debriefing with an external qualitative researcher, who had no involvement in the study and no clinical nursing background, was used as the principal external check. These sessions covered the coding framework, the developing thematic structure, and representative extracts, and the debriefer was asked specifically to argue against the emerging interpretation rather than to confirm it. Two of the interpretive moves in the final analysis, the reading of institutional non-response as a communicative act and the framing of resilience as compensation rather than achievement, were sharpened in response to challenges raised in these sessions.

During coding, accounts that did not fit the emerging framework were sought deliberately rather than set aside, and the question asked of each transcript was not only what supports the developing themes but what complicates them.

Eight participants reviewed a written summary of the preliminary findings. This returned interpretive authority to the people whose accounts were being analysed and provided a check that the analysis remained recognisable to them. Full transcripts were not returned.

Residual limitation

None of these strategies substitutes for a second analyst. Reflexive thematic analysis does not require one, and its developers argue that consensus coding misconstrues what the method is for, but the analysis presented here nonetheless reflects one researcher's sustained engagement with the data rather than a negotiated reading. This is stated in Section 4.6 of the manuscript, and readers should weigh the findings with it in view.
